# Postoperative examination after cataract surgery

**Published:** 2025-11-22

**Authors:** Lloyd Harrison-Williams

**Affiliations:** 1 Ophthalmologist/Programme Manager: National Eye Health Programme, Freetown, Sierra Leone.

**Even excellent surgery can result in a poor outcome if postoperative care is inadequate.**

**Figure:**
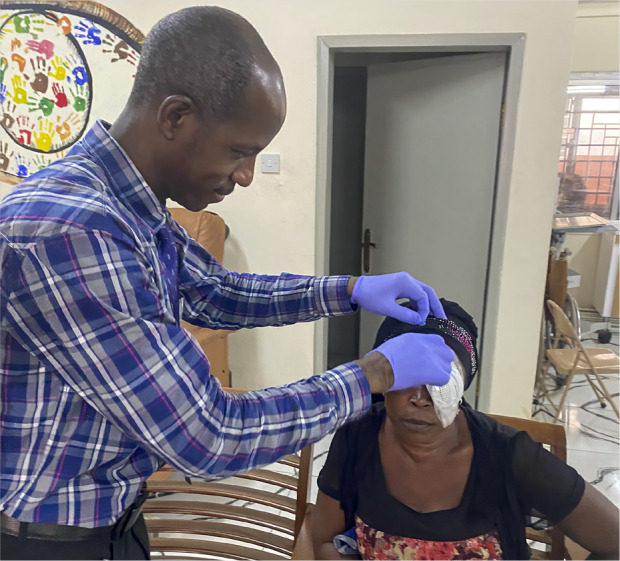
Preparing to examine a cataract patient after surgery. SIERRA LEONE

© LLOYD HARRISON-WILLIAMS CC BY-NC-SA 4.0

Performing a safe, high-quality operation is vital, but good postoperative care is arguably even more important. Complications can occur even in the best of hands and within the best ophthalmology institution. However, with careful postoperative management, the impact of those complications can be minimised and a good outcome achieved. On the contrary, even excellent surgery can result in a poor outcome if there isn't adequate postoperative care.

Examination of the postoperative eye commences at the time of removal of the eye patch, on postoperative day 1. The timing of further visits will depend on local context. Ideally, there should be three stages of follow-up, for the following reasons:

- **Day 1.** To identify complications, particularly those that are urgent and sight-threatening
- **Week 1.** To monitor healing, inflammation, and IOL position
- **Weeks 4–6.** To confirm stable healing, optimise vision (refraction), and detect late complications

**Note:** More frequent follow-up may be needed for high-risk patients (people with diabetes, patients with complex cataracts).

## What should be included in a postoperative assessment?

### Visual acuity

Check near and distance visual acuity (VA) (the latter with and without pinhole). Early assessment of both eyes after cataract surgery is important, even though it may not necessarily dictate the eventual postoperative unaided VA. The difference between unaided and pinhole VA may give an early indication of the extent of residual refractive error.

Early refraction will show if there has been a mismatch between the intraocular lens (IOL) power prescribed based on biometry measurement, and the actual IOL that was inserted. If this is the case, prompt surgery is helpful to exchange the IOL before the capsule fibroses. If this isn't possible, allow the remodelling phase of wound healing to elapse and the corneal oedema to settle before the residual refractive error can be assessed and adequately corrected.

**Who should carry out postoperative examination?**

It is essential that the person doing the postoperative examination knows the difference between acceptable levels of postoperative inflammation or swelling, and that which may signify a serious eye condition, which would necessitate hospital admission and intensive treatment with antibiotic and anti-inflammatory medications.

Postoperative examination is also an opportunity to identify any surgical causes of complications, which is helpful feedback for the surgeon. It is therefore highly recommended that surgeons should make it their point of duty to examine postoperative patients, especially during training or early in their career as a fully qualified surgeon.

### Intraocular pressure

Carefully check intraocular pressure (IOP), avoiding excess pressure on the globe. If excessively high (>30 mmHg), immediate treatment may be needed with acetazolamide and/or topical anti-glaucoma medications. If **retained viscoelastic** is the suspected cause, this can be let out at the slit lamp by carefully opening the paracentesis (if one was made during surgery). Regular monitoring of IOP should follow, until the pressure normalises and patient is weaned off the anti-glaucoma medications.

### Examination of the eyelids

If the eyelids are swollen, is there abnormal discharge? This may be an early sign of **postoperative infection**. If the eye is proptosed, then there may be **retrobulbar haemorrhage** from the anaesthetic injection. Urgently assess the optic nerve function to see if lateral canthotomy is needed.

### Examination of the conjunctiva

Is the conjunctiva extremely injected or red? Can you ascribe this to the sub-conjunctival or sub-Tenon's injection that was administered? If so, there is no reason to worry: the usual combination of antibiotic and steroid will help this to resolve. However, if this is associated with **discharge**, look for other signs of postoperative infection. Is there chemosis, eye pain, etc.? If so, it should be further investigated to rule out **endophthalmitis** or **anterior** **segment syndrome**, both of which are very serious. Check on other patients operated on that same day in case there is a cluster of the same complication.

### Examination of the cornea

This is also key in determining whether the unaided VA represents the likely long-term outcome. If the cornea is perfectly clear, with no endothelial folds and with a nicely centred posterior chamber IOL, a low unaided VA that improves with pinhole suggests some refractive error. Severe visual impairment that does not improve with pinhole in the presence of a reasonably clear cornea will most likely be due to **posterior segment pathology** such as vitreous haemorrhage, macular hole/scar, retinal detachment, or advanced glaucoma.

**Figure 1:**
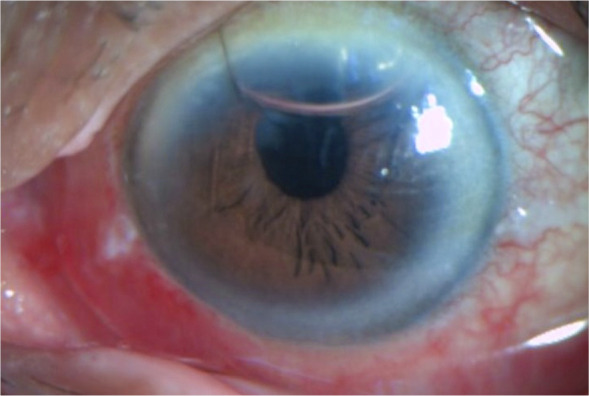
Corneal oedema after surgery.

Check for **corneal oedema** (Figure 1). This could indicate raised IOP, corneal touch during surgery, or viscoelastic that has not been adequately removed.

© LILA RAJ PURI CC BY-NC-SA 4.0

Assess **keratic precipitates** on the endothelium in view of the type of cataract that was operated, noting whether it was a uveitic or traumatic cataract.

Evaluate **corneal opacitie**s in view of previously documented corneal scarring. If there was no such history of corneal scar, determine intraoperative or immediate postoperative cause of the corneal complications. Intraoperative causes include Descemet's stripping, endothelial touch (especially during difficult nucleus delivery), or vitreous prolapse. Determining the cause will help in the treatment of the patient affected, and provides helpful feedback to the surgeon so they can prevent similar complications in future.

### Assessment of the surgical incision

Investigate any debris, blood clots, or potential evidence of prolapse of vitreous or iris from the corneoscleral  wound. Administer topical anaesthetic agents and use sterile cotton buds or eye spear sponges to swab away debris. Difficulty removing debris may suggest **vitreous prolapse**, revealed when simultaneous movement of the iris occurs when the cotton bud/sponge is used to swab the debris. A peaked pupil also substantiates vitreous, and/or **iris entrapment**. Both would need immediate surgery, paying special attention to maintaining sterility in order to prevent endophthalmitis.

**Figure 2:**
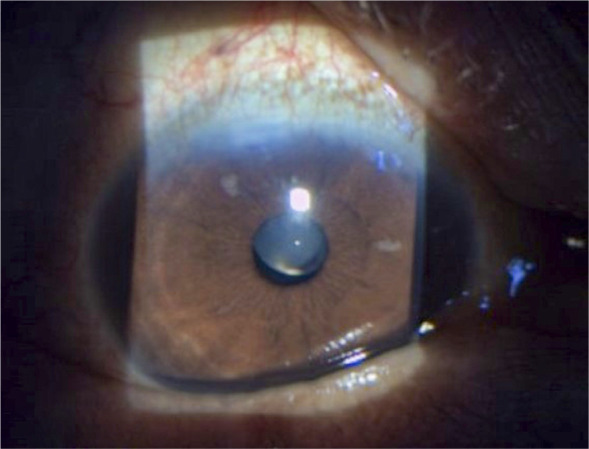
Clear cornea with IOL in bag and a healed incision.

On postoperative day 1, the incision is still covered by the conjunctiva. At 4–6 weeks postoperatively, the incision should be fully healed (Figure 2).

© LILA RAJ PURI CC BY-NC-SA 4.0

### Anterior chamber

**Figure 3:**
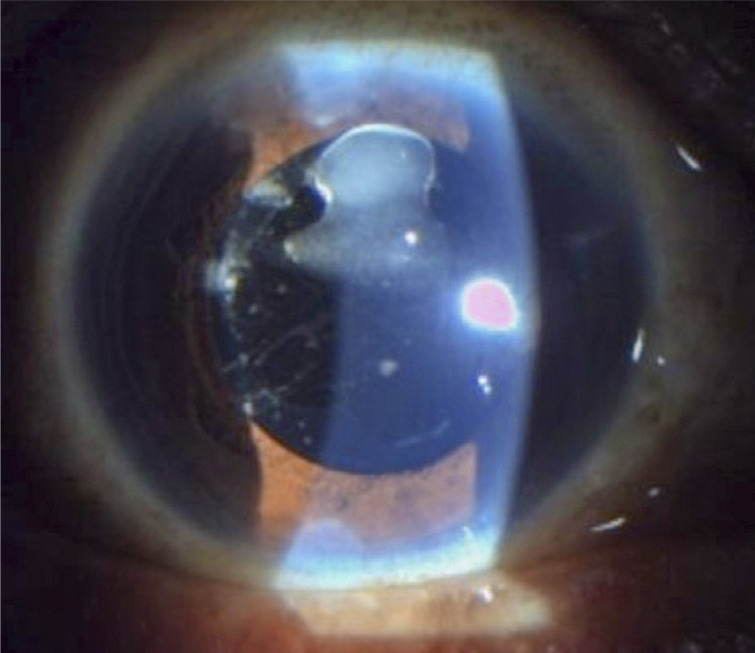
Cortical matter remaining in the anterior chamber. If it is significant in amount, and blocks the pupil or affects vision, it must be removed.

Assess the anterior chamber for cells and flare if corneal clarity permits, and for any retained cortical matter (Figure 3). **Note:** Air in the anterior chamber usually resolves after 2–3 days.

© LILA RAJ PURI CC BY-NC-SA 4.0

Patients with a **history of uveitis** should be identified preoperatively and treated with intensive preoperative steroids to ensure quiescence prior to surgery, then with ongoing intensive topical steroids postoperatively, with or without systemic steroids. Those without this history, who are found to have **postoperative uveitis**, may be treated with corticosteroid eyedrops such as prednisolone 1%. In addition to routine antibiotic eyedrops, mydriatic agents such as cyclopentolate 1% should be promptly commenced in adequate frequencies, then tapered in titration according to the severity of the anterior chamber activity. However, keep in mind the possibility of infection or uveitis in response to retained lens material. More frequent reviews of such patients are usually necessary and monitoring of their IOP is vital, particularly with prolonged steroid treatment, which can cause elevated pressure.

### Intraocular lens

If the unaided VA is low, then dilated examination is needed to look for a decentred or subluxated/luxated IOL. If this is the case, it is necessary to evaluate whether there is sufficient capsular support to allow a lens to be stable in the bag or sulcus, or if an alternative means of IOL fixation is needed.

### Fundus (if media are clear)

On the first postoperative day after surgery, perform a fundus examination to assess the retina and optic nerve. This makes it possible to detect issues such as retinal detachment, cystoid macular oedema, vitreous haemorrhage, or retained lens fragments. Early identification of these problems allows for prompt management, ensuring optimal visual outcomes and preventing long-term vision loss.

### Posterior capsular opacity

**Figure 4:**
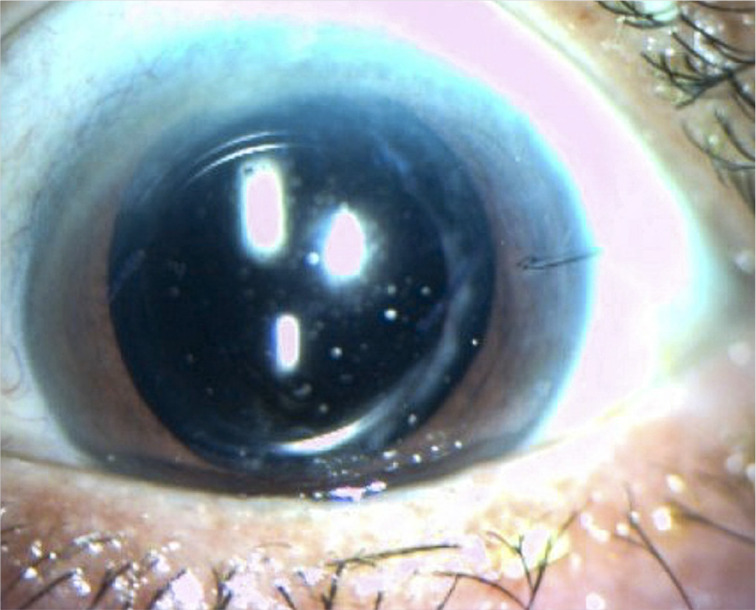
IOL in bag, mild postcapsular opacification, suture at side port.

This is a common postoperative complication, usually seen following dilation at the 4–6 week follow-up. If it significantly affects the patient's vision, then a YAG capsulotomy can easily be done to address it. If it is mild (Figure 4), it can be left as is.

© LILA RAJ PURI CC BY-NC-SA 4.0

### Communicating with patients

Talk to your patient, and the person accompanying them, about their visual prognosis and what treatment may be needed. This gives patients more clarity about what **they** will need to do to achieve the best possible visual outcome for them, such as coming back for further surgery, or instilling eye drops. Honest and open communication builds patients' trust in the health system, which in turn supports their willingness to adhere to prescribed management or medication.

